# Racial (In)Equity in South Los Angeles—Community Centered Experiences with COVID-19 Syndemics

**DOI:** 10.1089/heq.2023.0188

**Published:** 2024-07-01

**Authors:** Dilara K. Üsküp, Yelba M. Castellon-Lopez, Oluwadamilola Jolayemi, Cheryl A. Branch, Oladunni Adeyiga, Steve Shoptaw

**Affiliations:** ^1^ Department of Family Medicine, UCLA David Geffen School of Medicine, University of California, Los Angeles, California, USA.; ^2^ Department of Internal Medicine, Charles R. Drew University of Medicine, and Science, Los Angeles, California, USA.; ^3^ Department of Biomedical Sciences, Cedars-Sinai, Los Angeles, California, USA.; ^4^ The Community Response System of South Los Angeles (CRSSLA), Los Angeles, California, USA.; ^5^ Department of Medicine, Division of Infectious Diseases, UCLA David Geffen School of Medicine, Los Angeles, California, USA.

**Keywords:** COVID-19, health equity/disparities, coronavirus vaccination, vaccine hesitancy, minority health, community health

## Abstract

**Objectives:**

To analyze community experiences involving COVID-19 vaccination access and equity in Black and Latina/o/x communities within South Los Angeles, using a socioecological framework.

**Methods:**

We conducted four virtual focus groups (*n* = 33 total participants) in 2021, with Black and Latina/o/x community members, community leaders, and community-based providers in South Los Angeles, a region highly impacted by the COVID-19 pandemic. We used a grounded theory approach to guide the analysis and generate data shaped by participant perspectives.

**Results:**

Participants across groups consistently emphasized medical mistrust, fear/skepticism, misinformation, accessibility, and feelings of pressure and blame as factors influencing COVID-19 vaccination decisions. The need to address pandemic-related socioeconomic hardships in underresourced communities was equally highlighted.

**Conclusions:**

Findings show that building trust, providing tailored information, and continued investment into diversity and equity initiatives can support Black and Latino/a/x communities in making informed health decisions. Community-centered support services should address the economic, social, and structural impact of the pandemic on vulnerable communities. Furthermore, public health and policy efforts must prioritize funding to equip social and health care systems with infrastructure investment in racial and ethnic minority communities.

## Introduction

Like many urban communities across the nation, deaths in Los Angeles County due to SARS-CoV-2 infection throughout the COVID-19 pandemic concentrated among individuals from Black and Latina/o/x backgrounds, defining the disproportionate burdens to community, physical, mental, and economic health.^1,2^ Across the nation, evident disparities in hospitalizations and mortality rates persisted among Black and Latina/o/x communities.^3–8^ In April 2020, Centers for Disease Control and Prevention (CDC) data reported that African American populations, although only 13% of the US population made up 33% of patients hospitalized for COVID-19, compared with White populations accounting for 45% of patients hospitalized, though 76% of the US population.^9,10^ California similarly reported disproportionate rates of hospitalizations and deaths, with Latina/o/x populations overrepresented in reported cases, hospitalizations, and mortality from COVID-19.^8^ In Los Angeles County, as in other areas of the United States, as disproportionate deaths accrued, the unwillingness of people from Black and Latina/o/x racial and ethnic backgrounds to get vaccinated against COVID-19 presented a challenge.^11^ Studies have shown that multiple systemic barriers impacted vaccine uptake, including medical mistrust, lack of access to reliable and trusted information, problematic political messaging, and accessibility issues (lack of transportation and access to technology to make appointments).^12–14^ Residents of ethnic and racial minority communities also expressed frustrations ranging from concerns that individuals from highly resourced communities were traveling into lower resourced communities to “cut in line” and take doses intended for their community.^15,16^

In communities most impacted in Los Angeles County, such as South Los Angeles, multiple health threats already existed such as high levels of poverty, limited access to health care along with health insurance, high prevalence of chronic health conditions, multigenerational homes, overrepresentation in the frontline and essential workforce^1^, and lack of the privilege to work from home.^8,17–20^ These intersecting factors increased susceptibility to and occurrence of severe COVID-19 disease and its health and social implications in the area.^17,18^ Furthermore, racism, discrimination and inequity in the justice system coupled with historical mistreatment of Black and Latino patients by the health system increased negative impacts of the pandemic. This is especially the case within Black communities who were also faced with unprecedented social-political events at the onset of the pandemic. Tensions from the highly publicized deaths of young Black Americans—including George Floyd, Breonna Taylor, and Ahmaud Arbery—from racist actions of people representing systems that are charged with ensuring safety—further ignited government mistrust and greater attention to disparities, medical mistrust, and increased calls for health equity.

To inform policy decisions through elevating community experiences in real-time, our study sought to understand the factors that impacted COVID-19 vaccine rollout and uptake in South Los Angeles, particularly highlighting how an interplay of these factors could be used to improve vaccine equity in historically marginalized communities. Using qualitative methods, we sought to systematically gather perspectives and experiences around COVID-19 vaccination access and equity from members of various sectors of the South Los Angeles community. We report our findings using the socioecological model. This framework considers the complex interplay between factors at the individual (intrapersonal), interpersonal (relationships), community (physical/social environment), and societal (structures/systems) levels, and how these factors influence health.^21^ It allows us to understand the range of factors that put people at risk for poor outcomes and how to approach health interventions from a comprehensive approach, considering multilevels of influence. We invited perspectives from individuals who identified as community members, community leaders, and community clinicians in South Los Angeles, an area overwhelmed by increased disease burden, poverty, and violence.

## Methods

We conducted four virtual focus groups with participants (*n* = 33) from June 2021 to August 2021 to gather perspectives about the distribution of vaccines within Black and Latina/o/x communities in South Los Angeles. See Table 1. Our four focus groups used purposeful sampling to explore the perspectives of three key groups: community members, community leaders, and community clinicians. Specifically focusing on groups that were disproportionately impacted by the COVID-19 pandemic, the first two focus groups, conducted in English and in Spanish, included community members, such as essential workers, parents, and community stakeholders. The third focus group included community leaders such as faith-based leaders and leaders of community-based organizations, and the fourth comprised of Black and Latina/o/x community clinicians living in and/or providing services in South Los Angeles. This study was reviewed and approved by the Institutional Review Board (IRB #21–000339).

**Table 1.** Baseline Demographic Characteristics of Sample

Sociodemographic characteristics	Total *n* = 33
Groups	*n* (%)
Community members (English-speaking)	6 (18)
Community members (Spanish-speaking)	10 (30)
Community leaders	11 (33)
Community clinical providers	6 (18)
Age	(SD, M, Range) (13.59, 46.3 years, 19–83 years)
Sex assigned at birth	*n* (%)
Female	20 (61)
Male	12 (36)
No response	1 (3)
Gender identity	*n* (%)
Female	20 (61)
Male	11 (33)
Transgender female/woman	1 (3)
No response	1 (3)
Race/ethnicity	n (%)
American Indian/Alaska Native (Hispanic/Latino/a/x)	1 (3)
Asian	1 (3)
Black (Non-Hispanic/Latinx)	16 (48)
Black (not identified)	3 (9)
White (Hispanic/Latino/a/x)	7 (21)
White (non-Hispanic/Latinx)	1 (3)
Other (Hispanic/Latinx)	1 (3)
Other/unidentified	3 (6)
Education	*n* (%)
Less than high school diploma	3 (9)
High school diploma or GED	8 (24)
Some college	8 (24)
Completed college/bachelor’s degree	7 (21)
Completed master’s degree	4 (12)
Completed doctorate PhD, MD, JD	2 (6)
No response	1 (3)
Vaccination status—the Pfizer, Moderna, or Johnson & Johnson vaccines	*n* (%)
Vaccinated	26 (79)
Unvaccinated	6 (18)
No response	1 (3)

Source/Notes: SOURCE (Authors’ sample data gathered during the study).

Flyers describing this research opportunity were distributed virtually to community partners and employers in South Los Angeles through social media and email. Eligibility criteria included adults aged 18 or older who identified as Black-African, African American, Black Multiracial and/or Latina/o/x, who were community members, leaders, or clinicians in South Los Angeles. Interested individuals completed a phone screening, and the research team determined which participants met the eligibility criteria. Eligible participants were invited by email to participate in a virtual focus group on Zoom. Participants completed a brief sociodemographic survey and were provided with an information sheet and study overview before the commencement of the focus groups. Each of the focus groups was conducted by trained facilitators (Y.C.-L., O.A., D.Ü., and S.S.) for 90 min using a semi-structured focus group guide. The semi-structured guide included prompts about the impact of personal perceptions, relationships, the environment, and societal structures on the decision to take the COVID-19 vaccines. We used the WHO socioecological model framework and the understanding that medical mistrust operates as a social determinant of health to develop our semi-structured interview guide.^22^ Focus groups were recorded, transcribed by a member of the research team, and Spanish transcripts were translated by a native Spanish speaking member of the research team. Participants each received a $50 electronic Visa gift card for their time. Each focus group was digitally recorded, stored on a secure server, and transcribed for analysis.

Data analysis was an iterative process using constant comparative analysis. An initial codebook was established using the semi-structured focus group guide, along with a meticulous line-by-line review of the transcripts, and focus group notes.^23^ Three members of the research team (O.J., D.Ü., and S.S.) met weekly to review, and refine the codebook until a consensus was achieved among the trained coders. The coding process unfolded across multiple stages, with coders independently coding a subset of transcripts through open coding to identify emerging concepts and patterns. Through continuous comparison, codes were organized and classified into broader themes. Discrepancies in coding were led by the PI (D.Ü.) and resolved through discussion until consensus was reached.^24^ A thematic analysis approach was used, leveraging data from the separate focus groups to understand general perceptions of COVID-19 vaccines, determine barriers to vaccine uptake, and identify hope for the integration of COVID-19 prevention and care with other health issues.^24,25^ Themes were then categorized into the different levels of the socioecological framework based on defined categories and their level of influence on vaccine-related health outcomes.^21^ This framework facilitated the understanding of how various factors across different levels influenced vaccine acceptance within each focus group. Themes were further reviewed to ensure that they represented the most important and relevant components of the data noted by participants. The research team (Y.C.-L., O.J., D.Ü., S.S., O.A., and C.B.) collectively categorized all themes and conducted reanalysis until consensus was reached. See Table 2.

**Table 2.** Semi-Structured Interview Guide Prompts and Related Themes Categorized into Socioecological Levels

Socioecological framework	Example of focus group questions	Themes
Individual	What are your opinions about the COVID-19 vaccine(s)? ○ Do you think the vaccines are advantageous/ not advantageous? Why or why not? Would you be interested in taking the COVID-19 vaccines? Why or why not? Have you taken any of the COVID-19 vaccine(s)—the Pfizer, Moderna, or Johnson & Johnson vaccines? What are the main concerns that you have in regard to the COVID-19 vaccines?	Personal Beliefs Personal Experiences and Perceived Severity Perception of Vaccination Efforts Gender Specific Concerns Concern, Fear and Skepticism
Interpersonal	Have you heard any rumors about the vaccines? ○ What are some of the rumors you heard? Has anyone you know gotten sick with COVID-19 or died, and has that changed your opinion? Do you think people in your community are interested in taking the vaccines? ○ Why or why not? Among the people close to you, what proportion has taken the vaccines? What proportion would want you to take the vaccines or would not want you to take the vaccines?	Family Relationships
Community	Who should deliver the information about COVID-19 vaccines to your community? Have you heard any rumors about the vaccines? What are some of the rumors you heard? ○ Do you believe these rumors are true? Why or why not?	Misinformation and Falsehoods Messaging
Societal	Do you trust the COVID-19 vaccines? Why or why not? ○ In your family, have there been experiences where people have been mistreated by the medical profession? Have these experiences affected your view of the vaccines? Do you feel that the government is forthcoming with information about the COVID-19 vaccines? Do you feel that public health agencies (CDC, NIH, LAC DPH, and FDA) are forthcoming with information about the COVID-19 vaccines? Have you tried signing up for the vaccines? Do you know where to get the information to sign up for the vaccines i.e., phone number, website? How easy is it to sign up to get the vaccines?	Accessibility Medical Mistrust and Distrust Political Leadership Vaccination Policies and Strategies

CDC, Centers for Disease Control and Prevention; NIH, National Institutes of Health; LAC DPH, Los Angeles County Department of Public Health; FDA, Food and Drug Administration.

## Results

### Socioecological Framework and Qualitative Themes

Participant responses were organized across individual, interpersonal, community, and societal levels, indicating impact on motivating or deterring the uptake of vaccines in Black and Latina/o/x communities.

#### Individual

Participants shared that individual level factors such as personal beliefs, personal experiences, perceived severity of COVID-19, perception of vaccination efforts, gender-specific concerns, fear, skepticism, and uncertainty all played a role in framing decisions on COVID-19 vaccinations.

##### Personal beliefs

Participants had personal views about vaccinations in general that guided their decision on the COVID-19 vaccines.

*“I do not believe in vaccinations at all. I have never been vaccinated and neither have my children and family. We lost our parents at a very young age, and we decided to eat healthier, take natural herbs and take care of ourselves holistically.” (Community member-English speaking, Black Female, August 2021)*

##### Personal experiences and perceived severity of the virus

Community members indicated that their decision on the COVID-19 vaccines was influenced by their personal experiences and perception of the severity of the virus.

*“I took part in a clinical trial for the vaccine before it got approved. I participated because a lot of people had died. My family got COVID in Mexico. Everyone fell ill. My mom, my brother’s wife, my uncle all died. A lot of people that I know died in Mexico and here in Los Angeles. Too many people died. Besides, they gave incentives for you to get vaccinated. Those are the reasons why I got vaccinated.” (Community member-Spanish speaking, Male August 2021)*

##### Perception of vaccination efforts

Community participants negatively viewed some incentive strategies employed by the department of public health to improve vaccine uptake among community members as not genuine.

*“…and then on the other side you have in minority parks, they’re giving away headsets and all this stuff, trying to convince young black teens to take shots. It looks questionable. So, one side we’re seeing the results are scary. Then the other side we’re seeing that the people are trying to be bought. So even if it’s not true, even if we are healthy, even if the vaccination is … 100% amazing, it just looks weird. So, I think it’s more of the perception of it to me than anything else.” (Community member-English speaking, Black Male, August 2021)*

##### Gender-specific concerns

Some participants expressed concern around potential biological complications with the vaccines. A participant was particularly concerned with adverse reactions between the vaccines and silicone implants in transgender women.

*“…especially our black and brown trans community members, have been very hesitant when it comes to wanting to even obtain the vaccination…Especially our trans women, have, been under a process what they call as pumping. So, taking the silicone and getting, wider hips, more buttocks. And so, a lot of the [trans] community has been worried about, how that silicone will interact [within their bodies] if they got vaccinated … So, that’s just a lot of the feedback that I’ve gotten from both Black and Latin trans community members.” (Community leader, Black Transwoman, June 2021)*

Another participant expressed concern that women who are pregnant may need more information on the safety of vaccines for their unborn children.

*“You have these pregnant, women, people who are… Just don't understand enough. There isn't enough information…” (Community leader, Black Female, June 2021)*

*Concern, fear, and skepticism:* Participants indicated skepticism of the vaccines and uncertainty around the effectiveness of the vaccines as reasons to not get vaccinated. Community members felt it was better to delay vaccination (e.g., taking a “wait and see approach”) to observe adverse and side effects before making a final determination about vaccination.

*“…one of the areas of hesitancy, people were saying, I will wait till it’s either required or I will wait until the last possible minute so that I can be sure, you know, that if there are any symptoms that might occur, that I’d be aware of what they might be.” (Community leader, Black Female, June 2021)*

#### Interpersonal

Participants shared that some factors on the relationship level—with close peers, partners, and family members may influence their decision to take the COVID-19 vaccines.

##### Family relationships

Some participants stated that experience with family or community members who had severe COVID-19 influenced decisions to take the vaccine.

*“My family and I decided to get the vaccine because my brother and his family caught the virus, and my brother did get very ill. Thank God he didn't need to get hospitalized, but he did get very ill. He told me about all Covid symptoms he had – he couldn't breathe. Thank God we didn't get Covid, but I convinced my husband to get vaccinated after what happened to my brother.” (Community member—Spanish speaking, Latino Female, August 2021)*

Furthermore, the responsibility and desire to protect family members were major decision-making factors in favor of the vaccine.

*“… About the vaccine, I heard about children who had inflammation, like myocarditis, and that worries me a lot because I have a 14-year-old daughter, and I vaccinated her…”(Community member—Spanish speaking, Latino Female, August 2021)*

#### Community

Participants shared how their physical and social environments especially information and messaging about the vaccines within institutions, organizations, schools, workplaces, and neighborhoods impacted vaccination decisions.

##### Misinformation and falsehoods

Community members shared that vaccine rumors circulating within their communities and online networks influenced decisions around getting the vaccines.

“… *As the others have already mentioned, I live here around … which is mainly the Latino community, mostly people from Central America. And I don't want to sound racist or anything, but most of the people I've spoken to in my area refuse to get vaccinated because of the things you mentioned here – that the vaccines are microchips, that it’s the devil’s work, that the Government wants to control and sterilize us, etc” (Community member-Spanish speaking, Latino Male, August 2021)*

##### Messaging

Participants identified that receiving messaging around COVID-19 vaccines from trusted members of their communities is impactful on decision making and vaccine uptake.

*“’Cause it, we are the most trustworthy person of all black folks trust each other more than anything. Um, no nothing can convince us more than, than each other. So why we can't get the funding, it takes to get into our community and get the message to our people…” (Community member-English speaking, Black Male, August 2021)*

Furthermore, providers shared that the availability of trusted sources of information used as messaging tools to combat misinformation within the community may have an impact on vaccination decisions.

*“… So, we just kind of not create a narrative but we engage in this conversation as to why and where are you getting it, and then we point people to, you know, ad council, messaging. We point people to the CDC’s websites for patients and the public. And so we try to kind of present them with alternatives to their Twitter or Instagram, TikTok, Facebook sources and say, “But here is actually some information backed up with science.” And thankfully, here, locally, we've got some pretty good options with what UCLA, USC, [Charles] Drew. They've put together, trained the ambassador, messaging, PowerPoints that we use to train canvassing teams, outreach teams, so we really try to empower everyone around the vaccine program to have accurate information and then how to do this dance of, “Let’s walk back. You're not gonna grow a tail. We're not altering your DNA. We're not microchipping you. You're not gonna become a magnet.” (Community Provider, Other/Unidentified, August 2021)*

#### Societal

On the societal level, participants highlighted the impact societal factors such as structural inequity, political leadership, and policies and practices had on influencing vaccine uptake.

##### Accessibility

Participants expressed concern that the vaccines were not available to certain communities, also emphasizing technology barriers to scheduling appointments and lack of transportation to get to vaccination sites as factors influencing uptake.

*“I think it was at the beginning when they opened vaccination spots on the … stadium. You could get the vaccine in the parking lot and register online. We must remember that the virus hit the elderly the worst, and many of them live alone or don't know how to use a computer or do these types of things. It’s even more complicated for Latinos. They oversaw the issues with online registration, that there were problems with that, especially for the elderly. This is what I observed at the beginning.” (Community member-Spanish speaking, Latino Male, August 2021)*

##### Medical mistrust and distrust

Community members, community leaders, and clinical providers highlighted medical mistrust as one of the major reasons inhibiting decisions to take the COVID-19 vaccine. The historical context and collective memory of unethical medical experiments, denial of treatment, and existing health disparities were all cited as contributing to medical mistrust. The lack of trust in public health leaders, institutions, and conflicting public health messages were key reasons behind resistance to vaccination.

*“I think the most common thing that I hear from close people … [who are waiting to take the vaccine] … is the experiment that happened a long time ago with our parents and their parents and things like that. So, there’s a huge, huge fear. And I think as things started happening, which validated it, it kind of gives people this more of a reason to just stand and say, “I'm gonna wait or I'm not gonna take it at all.” (Community member-English speaking, Black Male, August 2021)*

*“… yes, historical reasons, yes… And people say because of, you know, past experimentation or denial of treatment or you know, false injections of things…and failure to really provide safe and effective care for Black communities. You know, the healthcare sector has a long history of gross offenses, particularly like in the areas of even maternal and infant health”. (Community leader, Black Female, June 2021)*

##### Political leadership

Participants expressed concern that the political rhetoric on vaccinations, especially at the national level, was chaotic, thereby breeding mistrust, which may have negatively influenced decisions to take the vaccines.

*“There was this societal split that was happening where the low-income and the homeless community or the minorities in general was looking at this bickering happen on the political side. So, you already felt ostracized. So then when you have, you know, Trump specifically saying one thing or, and then, you know, so you see at the very top, all of these kings fighting, you don’t know who to believe. So, you just simply go into the doors and protect your family. That’s what it feels like during the shot season is, you know what, we don’t know who to believe, ‘Let’s just go inside and protect our families.’ And that’s kind of, what it feels like is everybody’s just trying to protect their families because at the very top politically you saw the bickering you saw, ‘Oh, they’re lying.’ ‘They’re not, they’re telling the truth.’ ‘No, they’re lying.’ You know? And so, you just decided like any black family would, just protect yourself.” (Community member-English speaking, Black Male, August 2021)*

##### Vaccination policies and strategies

For some participants, vaccination strategies that employ blame for not getting vaccinations instead of recognizing the opportunity to provide tailored education for the unvaccinated was detrimental to vaccination uptake.

*“I think it should be more about educating. It shouldn’t be everybody [should] be forced to take a shot. They shouldn’t be asked to take a shot. I just think they should be saying, hey, um, just in case you don’t want to, based on your fears of the government, your mistrusts of the government, the history of the government, some of your underlying conditions, this is how you can stay healthy…” (Community member-English speaking, Black Male, August 2021)*

## Discussion

Our findings highlight the individual, interpersonal, community, and societal factors that independently and collectively impacted vaccine uptake in South Los Angeles. On the individual level, personal perceptions of the vaccination effort such as fear and skepticism shaped decisions around the COVID-19 vaccines and influenced (slowed) vaccine uptake in communities with minority racial and ethnic identities. Focusing on these individual factors is key to improving vaccine uptake in traditionally marginalized communities. Interpersonal factors based on relationship with one’s environment also influenced decisions to take the COVID-19 vaccine. We found that relationships and shared community experiences impacted behavior and decisions to vaccinate. Our findings show that shared experiences of medical mistrust and distrust in the system, as well as misinformation influenced decisions to seek prevention of COVID-19 through vaccination and even mask-wearing. Our findings are consistent with existing literature documenting the experience of various ethnic and racial minority communities, especially Black communities, who have historically endured discrimination and unethical practices in the research and health care system and are therefore prone to distrust of the health system.^13,14,26–28^

Furthermore, participants in our study reported the perceived speed in development of the currently available COVID-19 vaccines and approval for their use contributed to concerns and skepticism about the safety of vaccines. False information and disinformation disseminated especially on social media and online, also reduced vaccine confidence. These falsehoods tend to thrive in environments with existing skepticism and distrust of the health system.^29^ These factors influence future disease prevention efforts and must be addressed on a community level with strategies aimed at building trust and dispelling myths.

We found that community level factors can be addressed through partnerships with community organizations and trusted community leaders. These joint efforts between communities and federal/state-funded health and social agencies are extremely important. The role of partnerships with trusted community leaders and organizations in encouraging vaccination within their communities early on in the roll-out process cannot be overemphasized.^30^ Our participants reported that community leaders helped overcome barriers on the interpersonal level by modeling behavior and building trust through creative messaging that encouraged vaccination uptake and advocated for equitable access to vaccines.

Societal level factors help create an environment where vaccination is encouraged. Systemic racism and inequity impact health interventions and outcomes in various communities. It is critical to address these on the policy level to ensure access to equitable funding and health care.^31^ The COVID-19 pandemic exacerbated economic, social, and environmental threats to health that previously existed and still continue to a great extent. Communities who bore the major brunt of disease burden tend to lack disaster relief and crisis management support structures to respond to complex community needs, further marginalizing individuals from vulnerable and high-risk areas in Los Angeles.^32^

It’s crucial to recognize that various factors affecting vaccine uptake intersect at different levels—individual, interpersonal, community, and societal. Medical mistrust, rooted in historical and structural disparities, emerges as a salient factor influencing vaccine decisions. Amplified by personal experiences of discrimination, its impact was quite evident in the distrust of leadership and skepticism of the vaccines. Similarly, messaging about the vaccines cuts across all levels. Personal beliefs about the vaccines which impacted uptake were influenced strongly by the information available on the interpersonal, community and societal levels. To address these intersecting factors, innovative and multi-level approaches are necessary to combat the social determinants of health that COVID-19 exposed. Key public health and policy approaches include continuing investments in solid social and health care systems that emphasize “whole person” health. Also establishing policies that prioritize funding to equip community health care systems with infrastructure to hire and train trusted health messengers and to employ more medical providers who hold underrepresented identities to respond more competently to the unique needs of diverse communities and promote trust in the health care system. Moving forward, it is important to address the intersection of health and policy to ensure that future diseases are addressed but also to prepare for future pandemics and ongoing syndemics of structural racism, poverty, and inequity.

A key limitation of our study is that our outcomes reflect an urban county with access to certain funds and resources. Findings may not be generalizable to rural settings.

## Conclusion/Public Health Implications

This study highlights important considerations on the road to recovery from the COVID-19 pandemic and emphasizes the need to act on various levels to increase uptake of vaccines and to optimize prevention of future infectious diseases threats. Vaccines are currently accessible; however, uptake has plateaued.^33,34^ With the evolution of COVID-19 into an endemic disease, it is more imperative to continue addressing barriers to COVID-19 vaccination on all levels — individual, interpersonal, community, and societal, especially in vulnerable and hardly reached communities to ensure these communities are safe and thriving. With a robust structure in place that encompasses the complex interaction between these factors, we will be ready to address any future pandemic.

## Notes

1 Essential workers are workers that provide or support any necessary service (i.e., building supply workers, national security workers, and financial service workers)^19^ In health care, essential workers are defined as paid and unpaid persons serving in health care settings that have the potential for direct and indirect exposure to patients or infectious materials.^35^

## Abbreviations Used

%: Percentage
CDC: Centers for Disease Control and Prevention
COVID-19: Coronavirus disease 2019
FDA: Food and Drug Administration
GED: General Education Development
IRB: Institutional Review Board
JD: Juris Doctor
LAC DPH: Los Angeles County Department of Public Health
MD: Doctor of Medicine
N: Number
NIH: National Institutes of Health
PhD: Doctor of Philosophy
PI: Principal Investigator
SARS-CoV-2: Severe acute respiratory syndrome coronavirus 2
SD: Standard Deviation
WHO: World Health Organization
